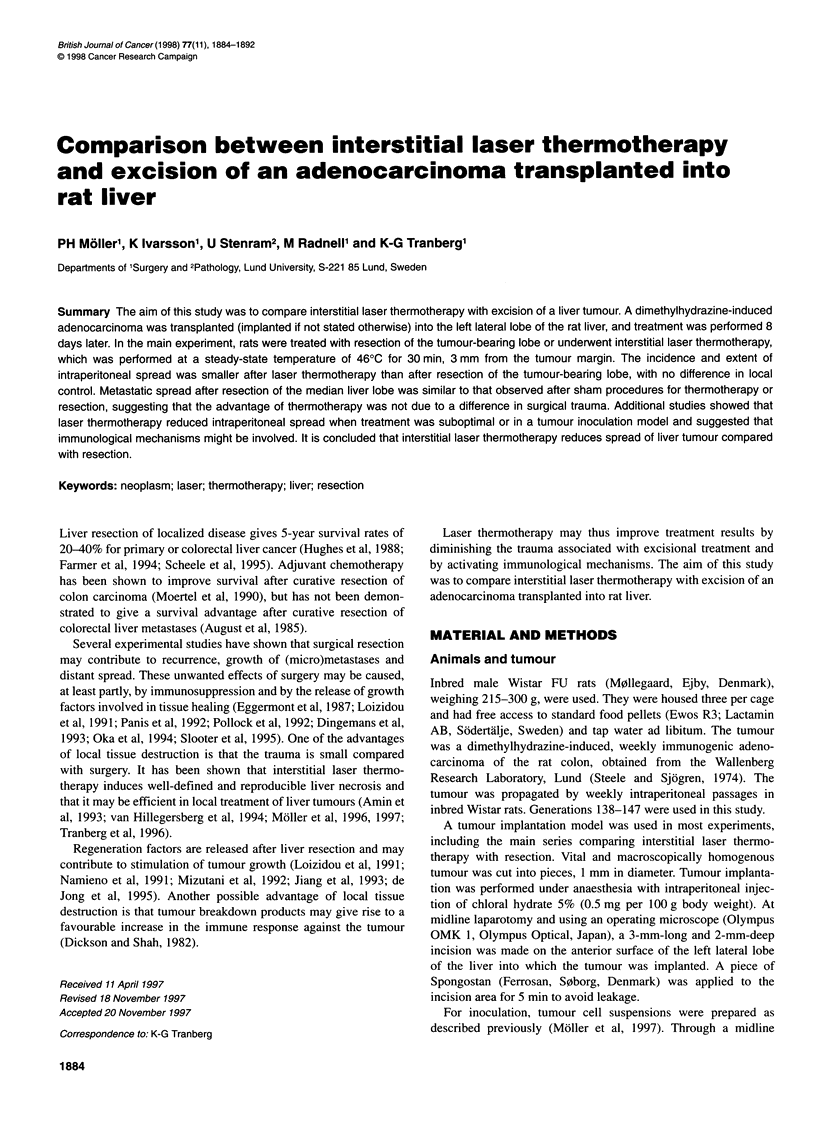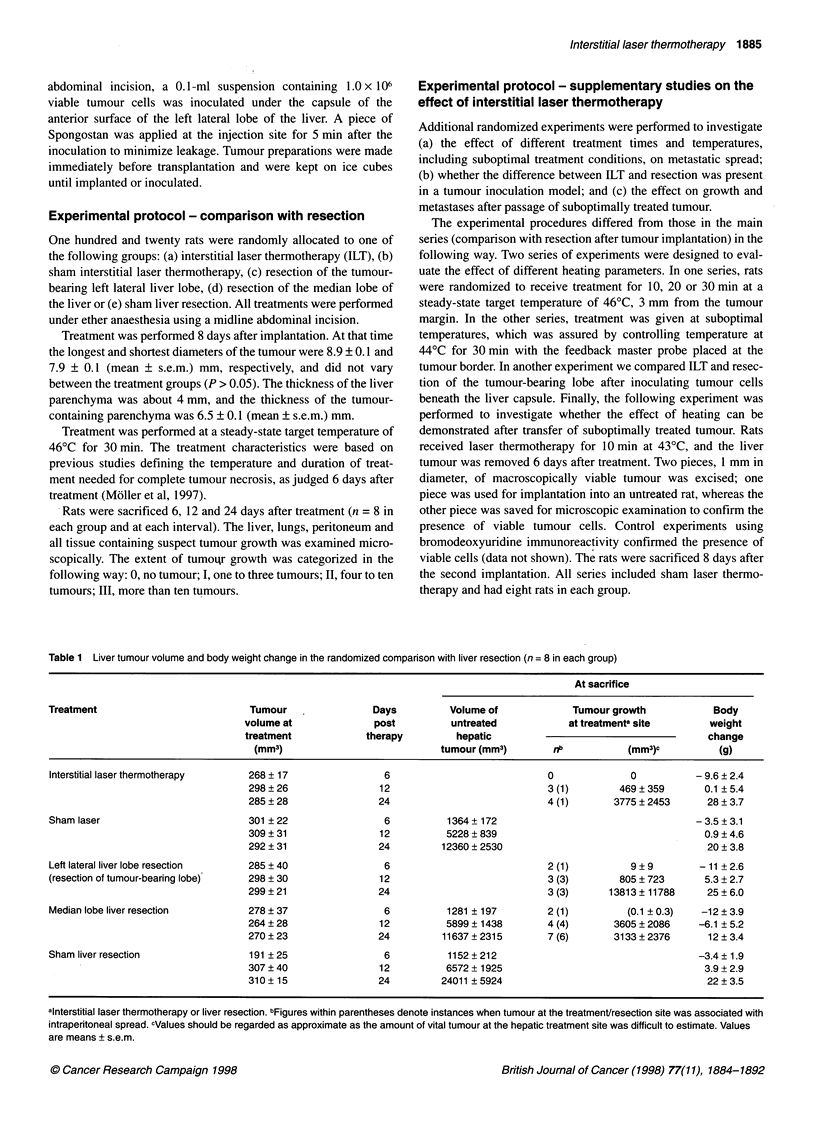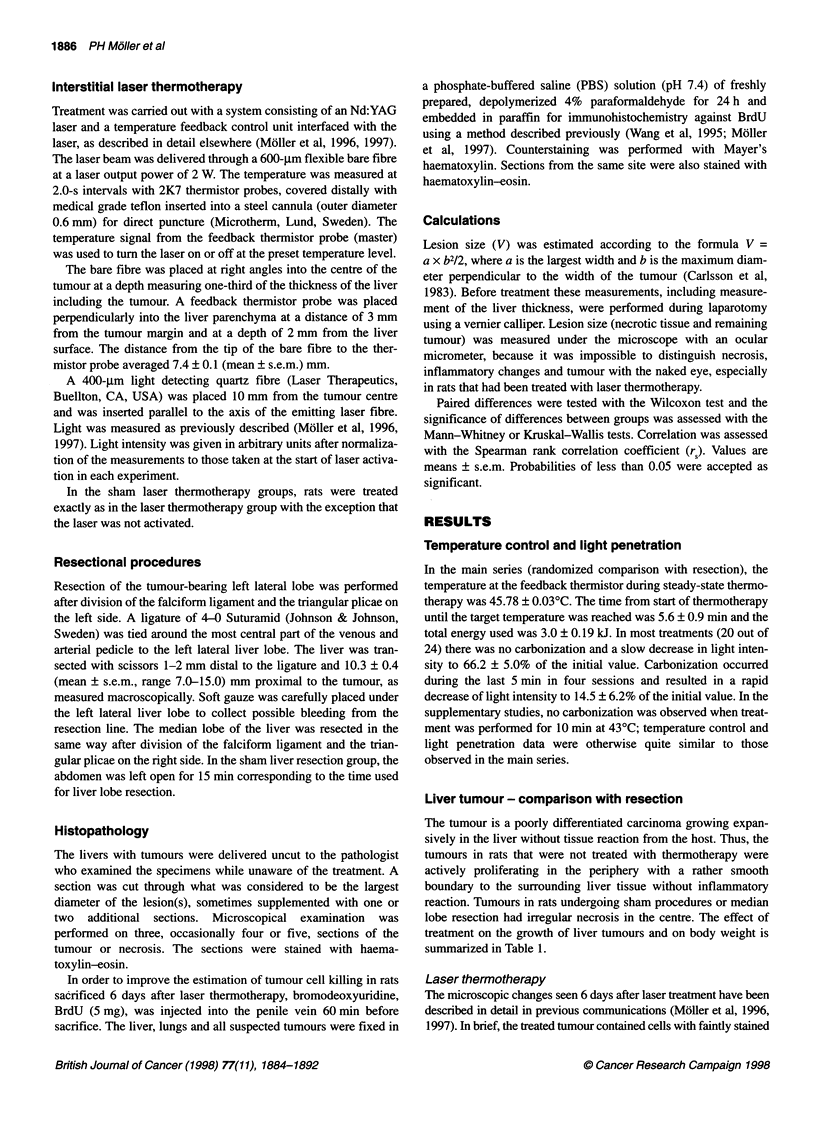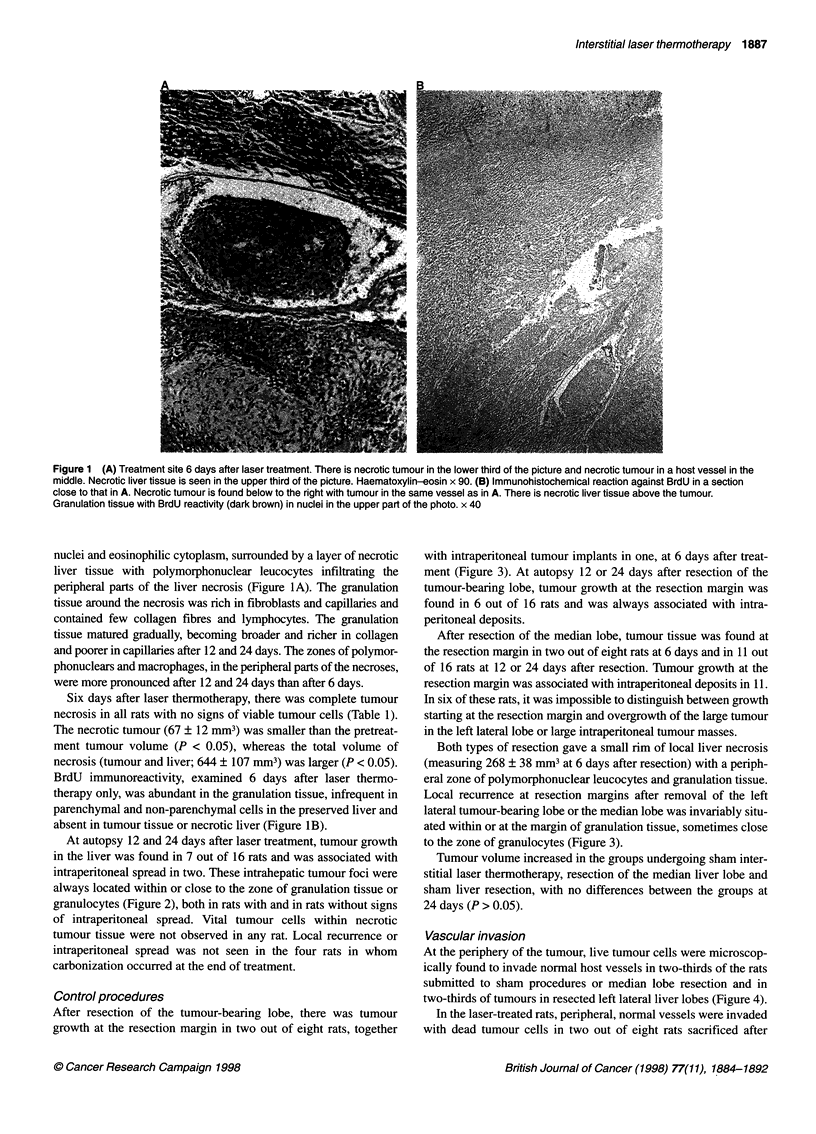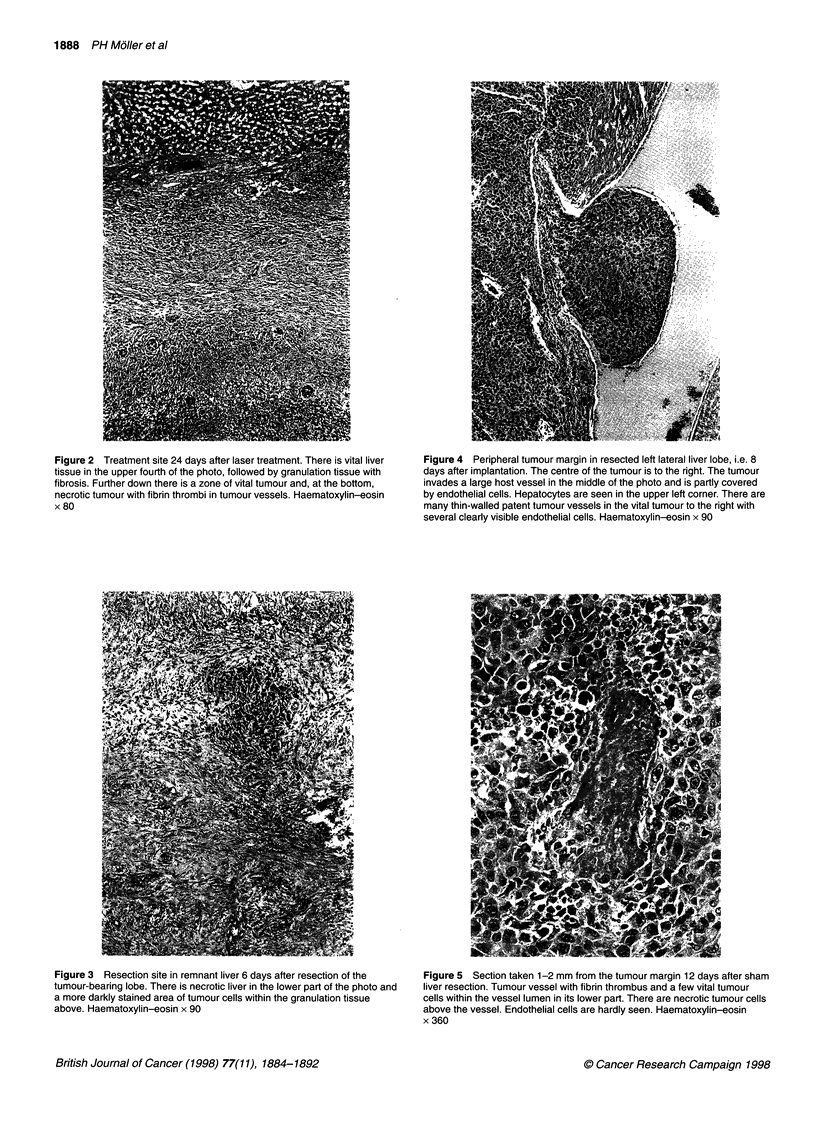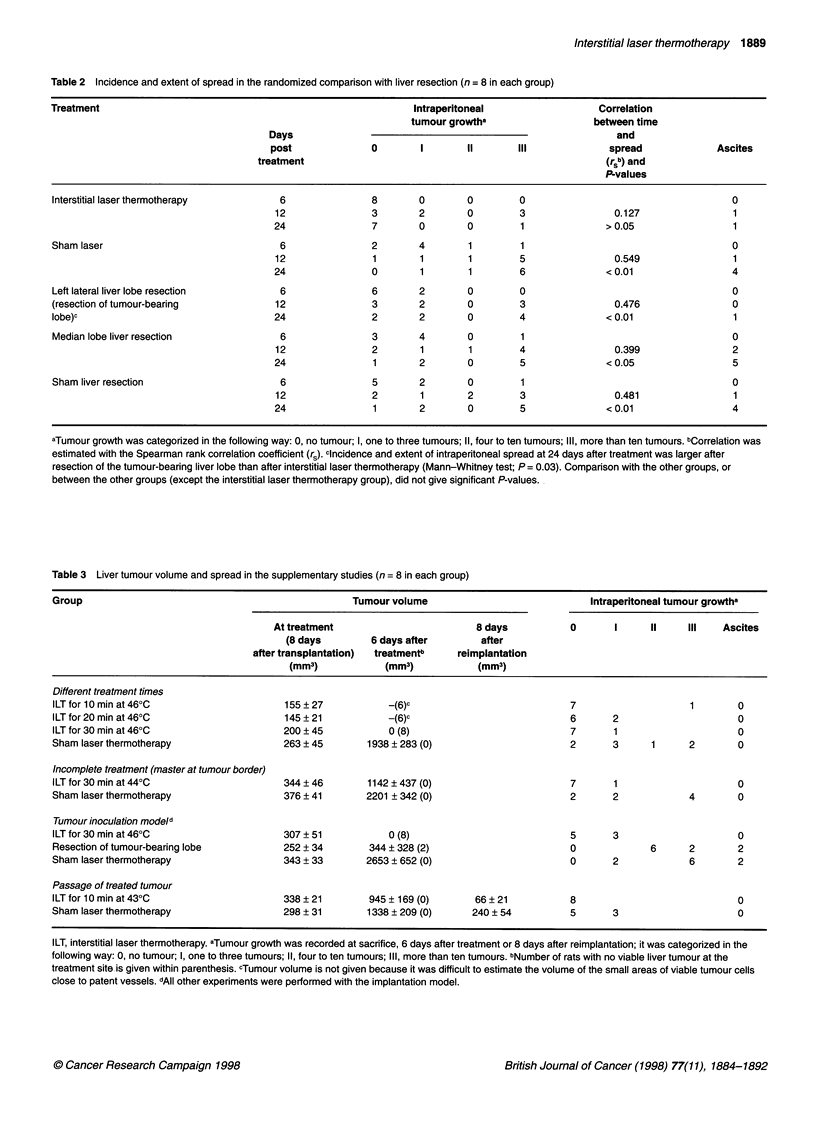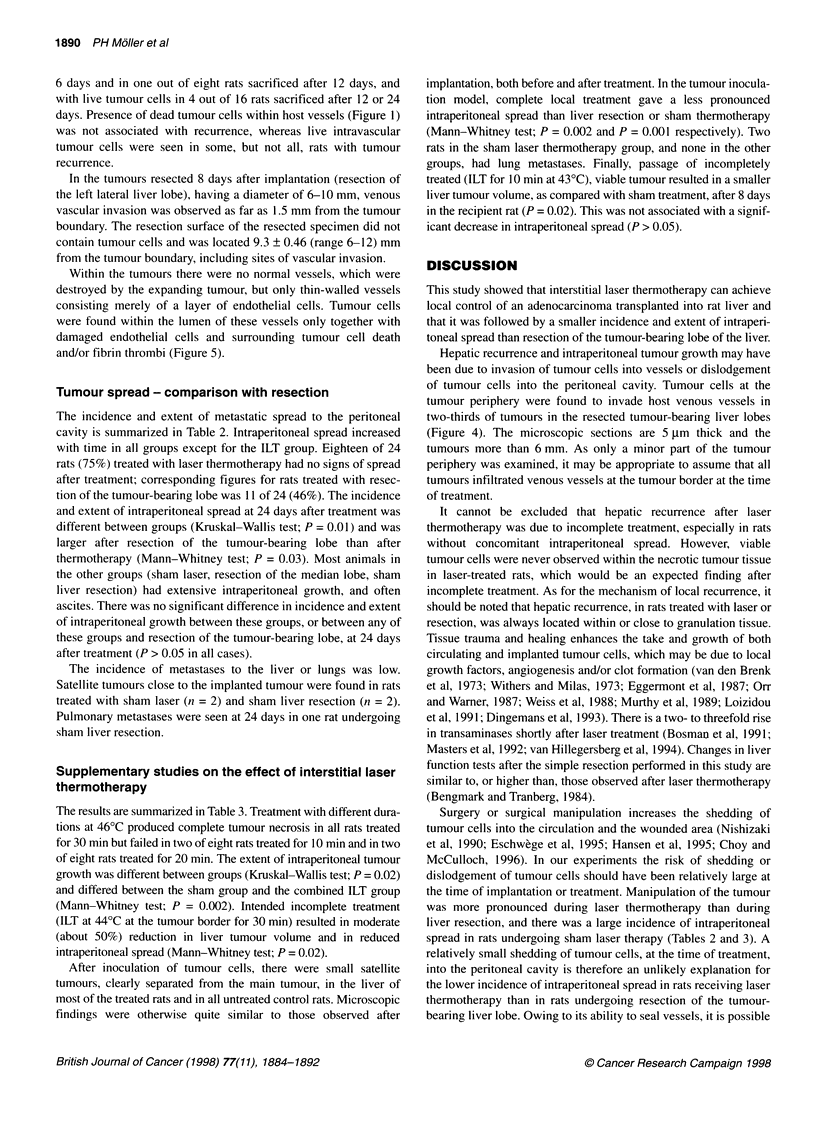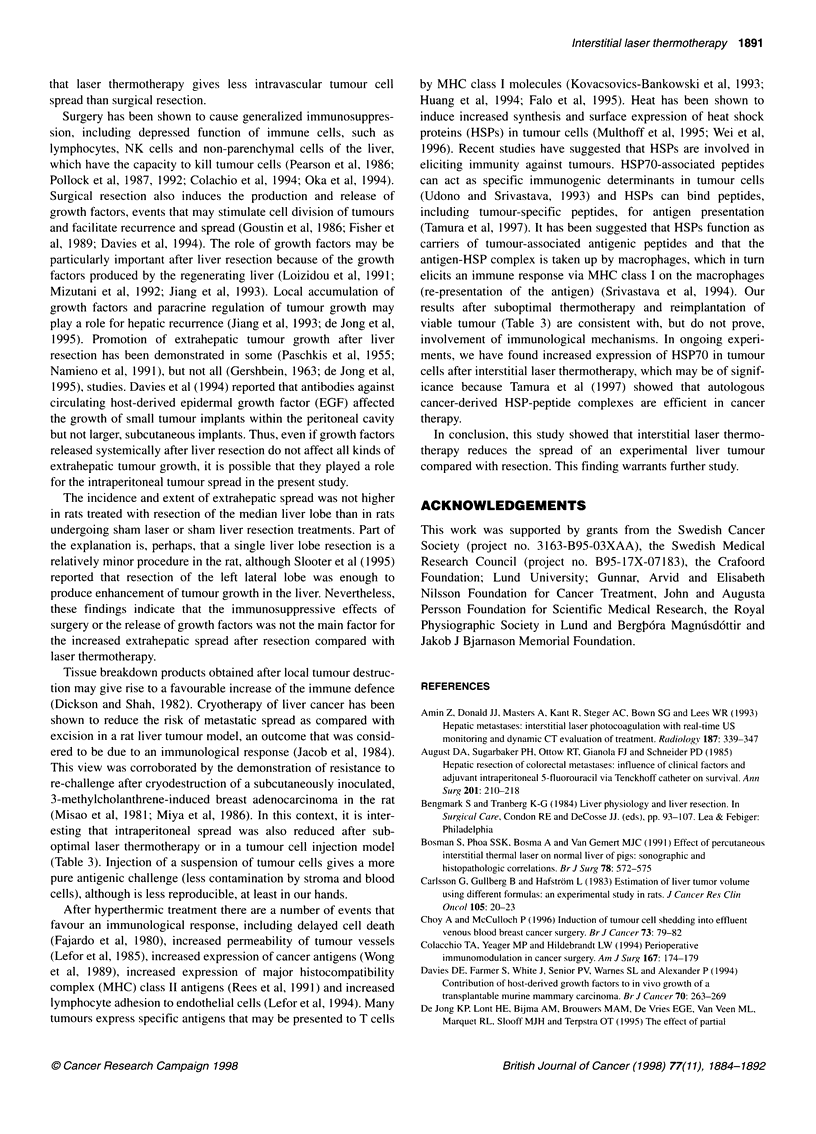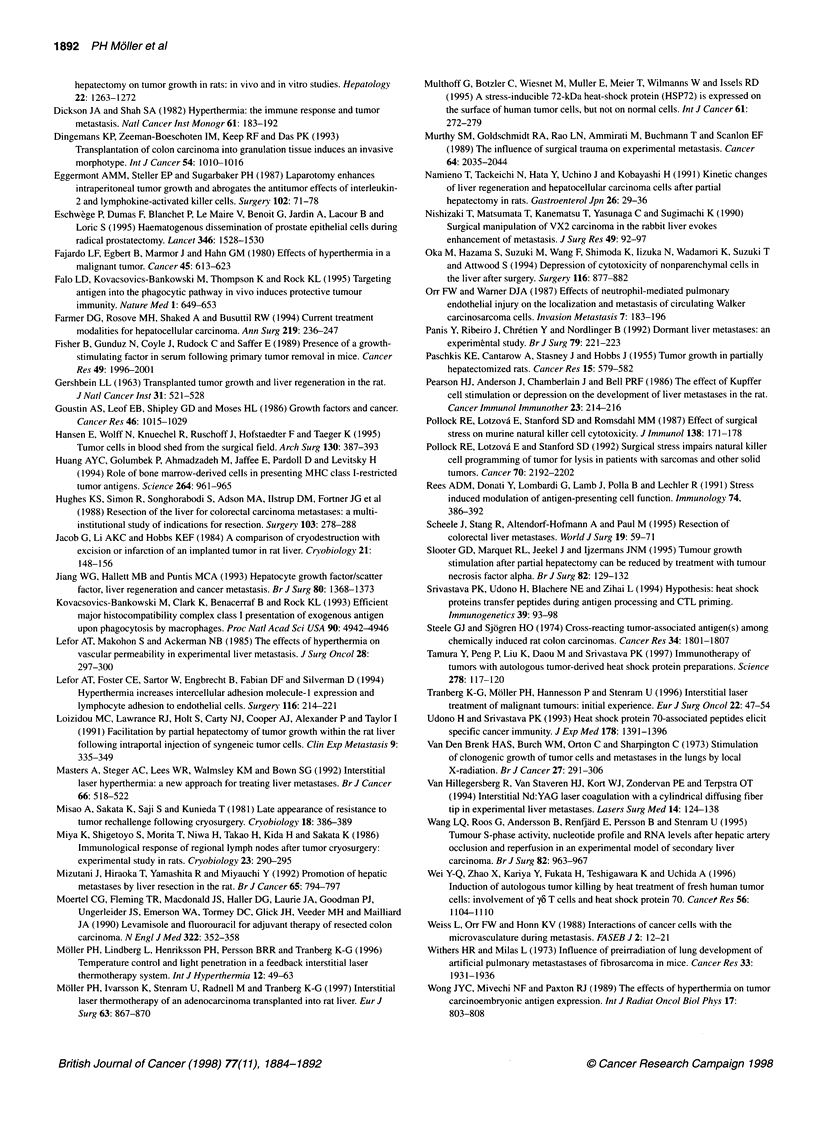# Comparison between interstitial laser thermotherapy and excision of an adenocarcinoma transplanted into rat liver.

**DOI:** 10.1038/bjc.1998.314

**Published:** 1998-06

**Authors:** P. H. MÃ¶ller, K. Ivarsson, U. Stenram, M. Radnell, K. G. Tranberg

**Affiliations:** Department of Surgery, Lund University, Sweden.

## Abstract

**Images:**


*British Journal of Cancer* (1998) **77**(11), 1884–1892
© 1998 Cancer Research Campaign

### Comparison between interstitial laser thermotherapy and excision of an adenocarcinoma transplanted into rat liver

**PH Möller[1], K Ivarsson[1], U Stenram[2], M Radnell[1] and K-G Tranberg[1]**

Departments of [1]Surgery and [2]Pathology, Lund University, S-221 85 Lund, Sweden

**Summary** The aim of this study was to compare interstitial laser thermotherapy with excision of a liver tumour. A dimethylhydrazine-induced adenocarcinoma was transplanted (implanted if not stated otherwise) into the left lateral lobe of the rat liver, and treatment was performed 8 days later. In the main experiment, rats were treated with resection of the tumour-bearing lobe or underwent interstitial laser thermotherapy, which was performed at a steady-state temperature of 46°C for 30 min, 3 mm from the tumour margin. The incidence and extent of intraperitoneal spread was smaller after laser thermotherapy than after resection of the tumour-bearing lobe, with no difference in local control. Metastatic spread after resection of the median liver lobe was similar to that observed after sham procedures for thermotherapy or resection, suggesting that the advantage of thermotherapy was not due to a difference in surgical trauma. Additional studies showed that laser thermotherapy reduced intraperitoneal spread when treatment was suboptimal or in a tumour inoculation model and suggested that immunological mechanisms might be involved. It is concluded that interstitial laser thermotherapy reduces spread of liver tumour compared with resection.

**Keywords:** neoplasm; laser; thermotherapy; liver; resection

Liver resection of localized disease gives 5-year survival rates of 20–40% for primary or colorectal liver cancer (Hughes et al, 1988; Farmer et al, 1994; Scheele et al, 1995). Adjuvant chemotherapy has been shown to improve survival after curative resection of colon carcinoma (Moertel et al, 1990), but has not been demonstrated to give a survival advantage after curative resection of colorectal liver metastases (August et al, 1985).

Several experimental studies have shown that surgical resection may contribute to recurrence, growth of (micro)metastases and distant spread. These unwanted effects of surgery may be caused, at least partly, by immunosuppression and by the release of growth factors involved in tissue healing (Eggermont et al, 1987; Loizidou et al, 1991; Panis et al, 1992; Pollock et al, 1992; Dingemans et al, 1993; Oka et al, 1994; Slooter et al, 1995). One of the advantages of local tissue destruction is that the trauma is small compared with surgery. It has been shown that interstitial laser thermotherapy induces well-defined and reproducible liver necrosis and that it may be efficient in local treatment of liver tumours (Amin et al, 1993; van Hillegersberg et al, 1994; Möller et al, 1996, 1997; Tranberg et al, 1996).

Regeneration factors are released after liver resection and may contribute to stimulation of tumour growth (Loizidou et al, 1991; Namieno et al, 1991; Mizutani et al, 1992; Jiang et al, 1993; de Jong et al, 1995). Another possible advantage of local tissue destruction is that tumour breakdown products may give rise to a favourable increase in the immune response against the tumour (Dickson and Shah, 1982).

*Received 11 April 1997*
*Revised 18 November 1997*
*Accepted 20 November 1997*

*Correspondence to:* K-G Tranberg

Laser thermotherapy may thus improve treatment results by diminishing the trauma associated with excisional treatment and by activating immunological mechanisms. The aim of this study was to compare interstitial laser thermotherapy with excision of an adenocarcinoma transplanted into rat liver.

### MATERIAL AND METHODS

### Animals and tumour

Inbred male Wistar FU rats (Møllegaard, Ejby, Denmark), weighing 215–300 g, were used. They were housed three per cage and had free access to standard food pellets (Ewos R3; Lactamin AB, Södertälje, Sweden) and tap water ad libitum. The tumour was a dimethylhydrazine-induced, weekly immunogenic adenocarcinoma of the rat colon, obtained from the Wallenberg Research Laboratory, Lund (Steele and Sjögren, 1974). The tumour was propagated by weekly intraperitoneal passages in inbred Wistar rats. Generations 138–147 were used in this study.

A tumour implantation model was used in most experiments, including the main series comparing interstitial laser thermotherapy with resection. Vital and macroscopically homogenous tumour was cut into pieces, 1 mm in diameter. Tumour implantation was performed under anaesthesia with intraperitoneal injection of chloral hydrate 5% (0.5 mg per 100 g body weight). At midline laparotomy and using an operating microscope (Olympus OMK 1, Olympus Optical, Japan), a 3-mm-long and 2-mm-deep incision was made on the anterior surface of the left lateral lobe of the liver into which the tumour was implanted. A piece of Spongostan (Ferrosan, Søborg, Denmark) was applied to the incision area for 5 min to avoid leakage.

For inoculation, tumour cell suspensions were prepared as described previously (Möller et al, 1997). Through a midline abdominal incision, a 0.1-ml suspension containing $1.0 \times 10^6$ viable tumour cells was inoculated under the capsule of the anterior surface of the left lateral lobe of the liver. A piece of Spongostan was applied at the injection site for 5 min after the inoculation to minimize leakage. Tumour preparations were made immediately before transplantation and were kept on ice cubes until implanted or inoculated.

### Experimental protocol – comparison with resection

One hundred and twenty rats were randomly allocated to one of the following groups: (a) interstitial laser thermotherapy (ILT), (b) sham interstitial laser thermotherapy, (c) resection of the tumour-bearing left lateral liver lobe, (d) resection of the median lobe of the liver or (e) sham liver resection. All treatments were performed under ether anaesthesia using a midline abdominal incision.

Treatment was performed 8 days after implantation. At that time the longest and shortest diameters of the tumour were 8.9 ± 0.1 and 7.9 ± 0.1 (mean ± s.e.m.) mm, respectively, and did not vary between the treatment groups ($P > 0.05$). The thickness of the liver parenchyma was about 4 mm, and the thickness of the tumour-containing parenchyma was 6.5 ± 0.1 (mean ± s.e.m.) mm.

Treatment was performed at a steady-state target temperature of 46°C for 30 min. The treatment characteristics were based on previous studies defining the temperature and duration of treatment needed for complete tumour necrosis, as judged 6 days after treatment (Möller et al, 1997).

Rats were sacrificed 6, 12 and 24 days after treatment ($n = 8$ in each group and at each interval). The liver, lungs, peritoneum and all tissue containing suspect tumour growth was examined microscopically. The extent of tumour growth was categorized in the following way: 0, no tumour; I, one to three tumours; II, four to ten tumours; III, more than ten tumours.

### Experimental protocol – supplementary studies on the effect of interstitial laser thermotherapy

Additional randomized experiments were performed to investigate (a) the effect of different treatment times and temperatures, including suboptimal treatment conditions, on metastatic spread; (b) whether the difference between ILT and resection was present in a tumour inoculation model; and (c) the effect on growth and metastases after passage of suboptimally treated tumour.

The experimental procedures differed from those in the main series (comparison with resection after tumour implantation) in the following way. Two series of experiments were designed to evaluate the effect of different heating parameters. In one series, rats were randomized to receive treatment for 10, 20 or 30 min at a steady-state target temperature of 46°C, 3 mm from the tumour margin. In the other series, treatment was given at suboptimal temperatures, which was assured by controlling temperature at 44°C for 30 min with the feedback master probe placed at the tumour border. In another experiment we compared ILT and resection of the tumour-bearing lobe after inoculating tumour cells beneath the liver capsule. Finally, the following experiment was performed to investigate whether the effect of heating can be demonstrated after transfer of suboptimally treated tumour. Rats received laser thermotherapy for 10 min at 43°C, and the liver tumour was removed 6 days after treatment. Two pieces, 1 mm in diameter, of macroscopically viable tumour was excised; one piece was used for implantation into an untreated rat, whereas the other piece was saved for microscopic examination to confirm the presence of viable tumour cells. Control experiments using bromodeoxyuridine immunoreactivity confirmed the presence of viable cells (data not shown). The rats were sacrificed 8 days after the second implantation. All series included sham laser thermotherapy and had eight rats in each group.

**Table 1** Liver tumour volume and body weight change in the randomized comparison with liver resection ($n = 8$ in each group)

| Treatment | Tumour volume at treatment (mm³) | Days post therapy | At sacrifice: Volume of untreated hepatic tumour (mm³) | Tumour growth at treatment[a] site: $n^b$ | Tumour growth at treatment[a] site: (mm³)[c] | Body weight change (g) |
|---|---|---|---|---|---|---|
| Interstitial laser thermotherapy | 268 ± 17 | 6 | | 0 | 0 | − 9.6 ± 2.4 |
| | 298 ± 26 | 12 | | 3 (1) | 469 ± 359 | 0.1 ± 5.4 |
| | 285 ± 28 | 24 | | 4 (1) | 3775 ± 2453 | 28 ± 3.7 |
| Sham laser | 301 ± 22 | 6 | 1364 ± 172 | | | − 3.5 ± 3.1 |
| | 309 ± 31 | 12 | 5228 ± 839 | | | 0.9 ± 4.6 |
| | 292 ± 31 | 24 | 12360 ± 2530 | | | 20 ± 3.8 |
| Left lateral liver lobe resection (resection of tumour-bearing lobe) | 285 ± 40 | 6 | | 2 (1) | 9 ± 9 | − 11 ± 2.6 |
| | 298 ± 30 | 12 | | 3 (3) | 805 ± 723 | 5.3 ± 2.7 |
| | 299 ± 21 | 24 | | 3 (3) | 13813 ± 11788 | 25 ± 6.0 |
| Median lobe liver resection | 278 ± 37 | 6 | 1281 ± 197 | 2 (1) | (0.1 ± 0.3) | −12 ± 3.9 |
| | 264 ± 28 | 12 | 5899 ± 1438 | 4 (4) | 3605 ± 2086 | −6.1 ± 5.2 |
| | 270 ± 23 | 24 | 11637 ± 2315 | 7 (6) | 3133 ± 2376 | 12 ± 3.4 |
| Sham liver resection | 191 ± 25 | 6 | 1152 ± 212 | | | −3.4 ± 1.9 |
| | 307 ± 40 | 12 | 6572 ± 1925 | | | 3.9 ± 2.9 |
| | 310 ± 15 | 24 | 24011 ± 5924 | | | 22 ± 3.5 |

[a]Interstitial laser thermotherapy or liver resection. [b]Figures within parentheses denote instances when tumour at the treatment/resection site was associated with intraperitoneal spread. [c]Values should be regarded as approximate as the amount of vital tumour at the hepatic treatment site was difficult to estimate. Values are means ± s.e.m.

© Cancer Research Campaign 1998

### Interstitial laser thermotherapy

Treatment was carried out with a system consisting of an Nd:YAG laser and a temperature feedback control unit interfaced with the laser, as described in detail elsewhere (Möller et al, 1996, 1997). The laser beam was delivered through a 600-μm flexible bare fibre at a laser output power of 2 W. The temperature was measured at 2.0-s intervals with 2K7 thermistor probes, covered distally with medical grade teflon inserted into a steel cannula (outer diameter 0.6 mm) for direct puncture (Microtherm, Lund, Sweden). The temperature signal from the feedback thermistor probe (master) was used to turn the laser on or off at the preset temperature level.

The bare fibre was placed at right angles into the centre of the tumour at a depth measuring one-third of the thickness of the liver including the tumour. A feedback thermistor probe was placed perpendicularly into the liver parenchyma at a distance of 3 mm from the tumour margin and at a depth of 2 mm from the liver surface. The distance from the tip of the bare fibre to the thermistor probe averaged 7.4 ± 0.1 (mean ± s.e.m.) mm.

A 400-μm light detecting quartz fibre (Laser Therapeutics, Buellton, CA, USA) was placed 10 mm from the tumour centre and was inserted parallel to the axis of the emitting laser fibre. Light was measured as previously described (Möller et al, 1996, 1997). Light intensity was given in arbitrary units after normalization of the measurements to those taken at the start of laser activation in each experiment.

In the sham laser thermotherapy groups, rats were treated exactly as in the laser thermotherapy group with the exception that the laser was not activated.

### Resectional procedures

Resection of the tumour-bearing left lateral lobe was performed after division of the falciform ligament and the triangular plicae on the left side. A ligature of 4–0 Suturamid (Johnson & Johnson, Sweden) was tied around the most central part of the venous and arterial pedicle to the left lateral liver lobe. The liver was transected with scissors 1–2 mm distal to the ligature and 10.3 ± 0.4 (mean ± s.e.m., range 7.0–15.0) mm proximal to the tumour, as measured macroscopically. Soft gauze was carefully placed under the left lateral liver lobe to collect possible bleeding from the resection line. The median lobe of the liver was resected in the same way after division of the falciform ligament and the triangular plicae on the right side. In the sham liver resection group, the abdomen was left open for 15 min corresponding to the time used for liver lobe resection.

### Histopathology

The livers with tumours were delivered uncut to the pathologist who examined the specimens while unaware of the treatment. A section was cut through what was considered to be the largest diameter of the lesion(s), sometimes supplemented with one or two additional sections. Microscopical examination was performed on three, occasionally four or five, sections of the tumour or necrosis. The sections were stained with haematoxylin–eosin.

In order to improve the estimation of tumour cell killing in rats sacrificed 6 days after laser thermotherapy, bromodeoxyuridine, BrdU (5 mg), was injected into the penile vein 60 min before sacrifice. The liver, lungs and all suspected tumours were fixed in a phosphate-buffered saline (PBS) solution (pH 7.4) of freshly prepared, depolymerized 4% paraformaldehyde for 24 h and embedded in paraffin for immunohistochemistry against BrdU using a method described previously (Wang et al, 1995; Möller et al, 1997). Counterstaining was performed with Mayer's haematoxylin. Sections from the same site were also stained with haematoxylin–eosin.

### Calculations

Lesion size ($V$) was estimated according to the formula $V = a \times b^2/2$, where $a$ is the largest width and $b$ is the maximum diameter perpendicular to the width of the tumour (Carlsson et al, 1983). Before treatment these measurements, including measurement of the liver thickness, were performed during laparotomy using a vernier calliper. Lesion size (necrotic tissue and remaining tumour) was measured under the microscope with an ocular micrometer, because it was impossible to distinguish necrosis, inflammatory changes and tumour with the naked eye, especially in rats that had been treated with laser thermotherapy.

Paired differences were tested with the Wilcoxon test and the significance of differences between groups was assessed with the Mann–Whitney or Kruskal–Wallis tests. Correlation was assessed with the Spearman rank correlation coefficient ($r_s$). Values are means ± s.e.m. Probabilities of less than 0.05 were accepted as significant.

### RESULTS

### Temperature control and light penetration

In the main series (randomized comparison with resection), the temperature at the feedback thermistor during steady-state thermotherapy was 45.78 ± 0.03°C. The time from start of thermotherapy until the target temperature was reached was 5.6 ± 0.9 min and the total energy used was 3.0 ± 0.19 kJ. In most treatments (20 out of 24) there was no carbonization and a slow decrease in light intensity to 66.2 ± 5.0% of the initial value. Carbonization occurred during the last 5 min in four sessions and resulted in a rapid decrease of light intensity to 14.5 ± 6.2% of the initial value. In the supplementary studies, no carbonization was observed when treatment was performed for 10 min at 43°C; temperature control and light penetration data were otherwise quite similar to those observed in the main series.

### Liver tumour – comparison with resection

The tumour is a poorly differentiated carcinoma growing expansively in the liver without tissue reaction from the host. Thus, the tumours in rats that were not treated with thermotherapy were actively proliferating in the periphery with a rather smooth boundary to the surrounding liver tissue without inflammatory reaction. Tumours in rats undergoing sham procedures or median lobe resection had irregular necrosis in the centre. The effect of treatment on the growth of liver tumours and on body weight is summarized in Table 1.

#### *Laser thermotherapy*

The microscopic changes seen 6 days after laser treatment have been described in detail in previous communications (Möller et al, 1996, 1997). In brief, the treated tumour contained cells with faintly stained

© Cancer Research Campaign 1998

**Figure 1** **(A)** Treatment site 6 days after laser treatment. There is necrotic tumour in the lower third of the picture and necrotic tumour in a host vessel in the middle. Necrotic liver tissue is seen in the upper third of the picture. Haematoxylin–eosin × 90. **(B)** Immunohistochemical reaction against BrdU in a section close to that in **A**. Necrotic tumour is found below to the right with tumour in the same vessel as in **A**. There is necrotic liver tissue above the tumour. Granulation tissue with BrdU reactivity (dark brown) in nuclei in the upper part of the photo. × 40

nuclei and eosinophilic cytoplasm, surrounded by a layer of necrotic liver tissue with polymorphonuclear leucocytes infiltrating the peripheral parts of the liver necrosis (Figure 1A). The granulation tissue around the necrosis was rich in fibroblasts and capillaries and contained few collagen fibres and lymphocytes. The granulation tissue matured gradually, becoming broader and richer in collagen and poorer in capillaries after 12 and 24 days. The zones of polymorphonuclears and macrophages, in the peripheral parts of the necroses, were more pronounced after 12 and 24 days than after 6 days.

Six days after laser thermotherapy, there was complete tumour necrosis in all rats with no signs of viable tumour cells (Table 1). The necrotic tumour (67 ± 12 $mm^3$) was smaller than the pretreatment tumour volume ($P < 0.05$), whereas the total volume of necrosis (tumour and liver; 644 ± 107 $mm^3$) was larger ($P < 0.05$). BrdU immunoreactivity, examined 6 days after laser thermotherapy only, was abundant in the granulation tissue, infrequent in parenchymal and non-parenchymal cells in the preserved liver and absent in tumour tissue or necrotic liver (Figure 1B).

At autopsy 12 and 24 days after laser treatment, tumour growth in the liver was found in 7 out of 16 rats and was associated with intraperitoneal spread in two. These intrahepatic tumour foci were always located within or close to the zone of granulation tissue or granulocytes (Figure 2), both in rats with and in rats without signs of intraperitoneal spread. Vital tumour cells within necrotic tumour tissue were not observed in any rat. Local recurrence or intraperitoneal spread was not seen in the four rats in whom carbonization occurred at the end of treatment.

### *Control procedures*

After resection of the tumour-bearing lobe, there was tumour growth at the resection margin in two out of eight rats, together with intraperitoneal tumour implants in one, at 6 days after treatment (Figure 3). At autopsy 12 or 24 days after resection of the tumour-bearing lobe, tumour growth at the resection margin was found in 6 out of 16 rats and was always associated with intraperitoneal deposits.

After resection of the median lobe, tumour tissue was found at the resection margin in two out of eight rats at 6 days and in 11 out of 16 rats at 12 or 24 days after resection. Tumour growth at the resection margin was associated with intraperitoneal deposits in 11. In six of these rats, it was impossible to distinguish between growth starting at the resection margin and overgrowth of the large tumour in the left lateral lobe or large intraperitoneal tumour masses.

Both types of resection gave a small rim of local liver necrosis (measuring 268 ± 38 $mm^3$ at 6 days after resection) with a peripheral zone of polymorphonuclear leucocytes and granulation tissue. Local recurrence at resection margins after removal of the left lateral tumour-bearing lobe or the median lobe was invariably situated within or at the margin of granulation tissue, sometimes close to the zone of granulocytes (Figure 3).

Tumour volume increased in the groups undergoing sham interstitial laser thermotherapy, resection of the median liver lobe and sham liver resection, with no differences between the groups at 24 days ($P > 0.05$).

### *Vascular invasion*

At the periphery of the tumour, live tumour cells were microscopically found to invade normal host vessels in two-thirds of the rats submitted to sham procedures or median lobe resection and in two-thirds of tumours in resected left lateral liver lobes (Figure 4).

In the laser-treated rats, peripheral, normal vessels were invaded with dead tumour cells in two out of eight rats sacrificed after

© Cancer Research Campaign 1998

**Figure 2** Treatment site 24 days after laser treatment. There is vital liver tissue in the upper fourth of the photo, followed by granulation tissue with fibrosis. Further down there is a zone of vital tumour and, at the bottom, necrotic tumour with fibrin thrombi in tumour vessels. Haematoxylin–eosin × 80

**Figure 4** Peripheral tumour margin in resected left lateral liver lobe, i.e. 8 days after implantation. The centre of the tumour is to the right. The tumour invades a large host vessel in the middle of the photo and is partly covered by endothelial cells. Hepatocytes are seen in the upper left corner. There are many thin-walled patent tumour vessels in the vital tumour to the right with several clearly visible endothelial cells. Haematoxylin–eosin × 90

**Figure 3** Resection site in remnant liver 6 days after resection of the tumour-bearing lobe. There is necrotic liver in the lower part of the photo and a more darkly stained area of tumour cells within the granulation tissue above. Haematoxylin–eosin × 90

**Figure 5** Section taken 1–2 mm from the tumour margin 12 days after sham liver resection. Tumour vessel with fibrin thrombus and a few vital tumour cells within the vessel lumen in its lower part. There are necrotic tumour cells above the vessel. Endothelial cells are hardly seen. Haematoxylin–eosin × 360

*© Cancer Research Campaign 1998*

**Table 2** Incidence and extent of spread in the randomized comparison with liver resection ($n$ = 8 in each group)

| Treatment | Days post treatment | Intraperitoneal tumour growth[a] | | | | Correlation between time and spread ($r_s$[b]) and *P*-values | Ascites |
|---|---|---|---|---|---|---|---|
| | | 0 | I | II | III | | |
| Interstitial laser thermotherapy | 6 | 8 | 0 | 0 | 0 | | 0 |
| | 12 | 3 | 2 | 0 | 3 | 0.127 | 1 |
| | 24 | 7 | 0 | 0 | 1 | > 0.05 | 1 |
| Sham laser | 6 | 2 | 4 | 1 | 1 | | 0 |
| | 12 | 1 | 1 | 1 | 5 | 0.549 | 1 |
| | 24 | 0 | 1 | 1 | 6 | < 0.01 | 4 |
| Left lateral liver lobe resection | 6 | 6 | 2 | 0 | 0 | | 0 |
| (resection of tumour-bearing | 12 | 3 | 2 | 0 | 3 | 0.476 | 0 |
| lobe)[c] | 24 | 2 | 2 | 0 | 4 | < 0.01 | 1 |
| Median lobe liver resection | 6 | 3 | 4 | 0 | 1 | | 0 |
| | 12 | 2 | 1 | 1 | 4 | 0.399 | 2 |
| | 24 | 1 | 2 | 0 | 5 | < 0.05 | 5 |
| Sham liver resection | 6 | 5 | 2 | 0 | 1 | | 0 |
| | 12 | 2 | 1 | 2 | 3 | 0.481 | 1 |
| | 24 | 1 | 2 | 0 | 5 | < 0.01 | 4 |

[a]Tumour growth was categorized in the following way: 0, no tumour; I, one to three tumours; II, four to ten tumours; III, more than ten tumours. [b]Correlation was estimated with the Spearman rank correlation coefficient ($r_s$). [c]Incidence and extent of intraperitoneal spread at 24 days after treatment was larger after resection of the tumour-bearing liver lobe than after interstitial laser thermotherapy (Mann–Whitney test; $P$ = 0.03). Comparison with the other groups, or between the other groups (except the interstitial laser thermotherapy group), did not give significant *P*-values.

**Table 3** Liver tumour volume and spread in the supplementary studies ($n$ = 8 in each group)

| Group | Tumour volume | | | Intraperitoneal tumour growth[a] | | | | |
|---|---|---|---|---|---|---|---|---|
| | At treatment (8 days after transplantation) (mm³) | 6 days after treatment[b] (mm³) | 8 days after reimplantation (mm³) | 0 | I | II | III | Ascites |
| *Different treatment times* | | | | | | | | |
| ILT for 10 min at 46°C | 155 ± 27 | –(6)[c] | | 7 | | | 1 | 0 |
| ILT for 20 min at 46°C | 145 ± 21 | –(6)[c] | | 6 | 2 | | | 0 |
| ILT for 30 min at 46°C | 200 ± 45 | 0 (8) | | 7 | 1 | | | 0 |
| Sham laser thermotherapy | 263 ± 45 | 1938 ± 283 (0) | | 2 | 3 | 1 | 2 | 0 |
| *Incomplete treatment (master at tumour border)* | | | | | | | | |
| ILT for 30 min at 44°C | 344 ± 46 | 1142 ± 437 (0) | | 7 | 1 | | | 0 |
| Sham laser thermotherapy | 376 ± 41 | 2201 ± 342 (0) | | 2 | 2 | | 4 | 0 |
| *Tumour inoculation model*[d] | | | | | | | | |
| ILT for 30 min at 46°C | 307 ± 51 | 0 (8) | | 5 | 3 | | | 0 |
| Resection of tumour-bearing lobe | 252 ± 34 | 344 ± 328 (2) | | 0 | | 6 | 2 | 2 |
| Sham laser thermotherapy | 343 ± 33 | 2653 ± 652 (0) | | 0 | 2 | | 6 | 2 |
| *Passage of treated tumour* | | | | | | | | |
| ILT for 10 min at 43°C | 338 ± 21 | 945 ± 169 (0) | 66 ± 21 | 8 | | | | 0 |
| Sham laser thermotherapy | 298 ± 31 | 1338 ± 209 (0) | 240 ± 54 | 5 | 3 | | | 0 |

ILT, interstitial laser thermotherapy. [a]Tumour growth was recorded at sacrifice, 6 days after treatment or 8 days after reimplantation; it was categorized in the following way: 0, no tumour; I, one to three tumours; II, four to ten tumours; III, more than ten tumours. [b]Number of rats with no viable liver tumour at the treatment site is given within parenthesis. [c]Tumour volume is not given because it was difficult to estimate the volume of the small areas of viable tumour cells close to patent vessels. [d]All other experiments were performed with the implantation model.

6 days and in one out of eight rats sacrificed after 12 days, and with live tumour cells in 4 out of 16 rats sacrificed after 12 or 24 days. Presence of dead tumour cells within host vessels (Figure 1) was not associated with recurrence, whereas live intravascular tumour cells were seen in some, but not all, rats with tumour recurrence.

In the tumours resected 8 days after implantation (resection of the left lateral liver lobe), having a diameter of 6–10 mm, venous vascular invasion was observed as far as 1.5 mm from the tumour boundary. The resection surface of the resected specimen did not contain tumour cells and was located 9.3 ± 0.46 (range 6–12) mm from the tumour boundary, including sites of vascular invasion.

Within the tumours there were no normal vessels, which were destroyed by the expanding tumour, but only thin-walled vessels consisting merely of a layer of endothelial cells. Tumour cells were found within the lumen of these vessels only together with damaged endothelial cells and surrounding tumour cell death and/or fibrin thrombi (Figure 5).

### Tumour spread – comparison with resection

The incidence and extent of metastatic spread to the peritoneal cavity is summarized in Table 2. Intraperitoneal spread increased with time in all groups except for the ILT group. Eighteen of 24 rats (75%) treated with laser thermotherapy had no signs of spread after treatment; corresponding figures for rats treated with resection of the tumour-bearing lobe was 11 of 24 (46%). The incidence and extent of intraperitoneal spread at 24 days after treatment was different between groups (Kruskal–Wallis test; $P = 0.01$) and was larger after resection of the tumour-bearing lobe than after thermotherapy (Mann–Whitney test; $P = 0.03$). Most animals in the other groups (sham laser, resection of the median lobe, sham liver resection) had extensive intraperitoneal growth, and often ascites. There was no significant difference in incidence and extent of intraperitoneal growth between these groups, or between any of these groups and resection of the tumour-bearing lobe, at 24 days after treatment ($P > 0.05$ in all cases).

The incidence of metastases to the liver or lungs was low. Satellite tumours close to the implanted tumour were found in rats treated with sham laser ($n = 2$) and sham liver resection ($n = 2$). Pulmonary metastases were seen at 24 days in one rat undergoing sham liver resection.

### Supplementary studies on the effect of interstitial laser thermotherapy

The results are summarized in Table 3. Treatment with different durations at 46°C produced complete tumour necrosis in all rats treated for 30 min but failed in two of eight rats treated for 10 min and in two of eight rats treated for 20 min. The extent of intraperitoneal tumour growth was different between groups (Kruskal–Wallis test; $P = 0.02$) and differed between the sham group and the combined ILT group (Mann–Whitney test; $P = 0.002$). Intended incomplete treatment (ILT at 44°C at the tumour border for 30 min) resulted in moderate (about 50%) reduction in liver tumour volume and in reduced intraperitoneal spread (Mann–Whitney test; $P = 0.02$).

After inoculation of tumour cells, there were small satellite tumours, clearly separated from the main tumour, in the liver of most of the treated rats and in all untreated control rats. Microscopic findings were otherwise quite similar to those observed after implantation, both before and after treatment. In the tumour inoculation model, complete local treatment gave a less pronounced intraperitoneal spread than liver resection or sham thermotherapy (Mann–Whitney test; $P = 0.002$ and $P = 0.001$ respectively). Two rats in the sham laser thermotherapy group, and none in the other groups, had lung metastases. Finally, passage of incompletely treated (ILT for 10 min at 43°C), viable tumour resulted in a smaller liver tumour volume, as compared with sham treatment, after 8 days in the recipient rat ($P = 0.02$). This was not associated with a significant decrease in intraperitoneal spread ($P > 0.05$).

### DISCUSSION

This study showed that interstitial laser thermotherapy can achieve local control of an adenocarcinoma transplanted into rat liver and that it was followed by a smaller incidence and extent of intraperitoneal spread than resection of the tumour-bearing lobe of the liver.

Hepatic recurrence and intraperitoneal tumour growth may have been due to invasion of tumour cells into vessels or dislodgement of tumour cells into the peritoneal cavity. Tumour cells at the tumour periphery were found to invade host venous vessels in two-thirds of tumours in the resected tumour-bearing liver lobes (Figure 4). The microscopic sections are 5 μm thick and the tumours more than 6 mm. As only a minor part of the tumour periphery was examined, it may be appropriate to assume that all tumours infiltrated venous vessels at the tumour border at the time of treatment.

It cannot be excluded that hepatic recurrence after laser thermotherapy was due to incomplete treatment, especially in rats without concomitant intraperitoneal spread. However, viable tumour cells were never observed within the necrotic tumour tissue in laser-treated rats, which would be an expected finding after incomplete treatment. As for the mechanism of local recurrence, it should be noted that hepatic recurrence, in rats treated with laser or resection, was always located within or close to granulation tissue. Tissue trauma and healing enhances the take and growth of both circulating and implanted tumour cells, which may be due to local growth factors, angiogenesis and/or clot formation (van den Brenk et al, 1973; Withers and Milas, 1973; Eggermont et al, 1987; Orr and Warner, 1987; Weiss et al, 1988; Murthy et al, 1989; Loizidou et al, 1991; Dingemans et al, 1993). There is a two- to threefold rise in transaminases shortly after laser treatment (Bosman et al, 1991; Masters et al, 1992; van Hillegersberg et al, 1994). Changes in liver function tests after the simple resection performed in this study are similar to, or higher than, those observed after laser thermotherapy (Bengmark and Tranberg, 1984).

Surgery or surgical manipulation increases the shedding of tumour cells into the circulation and the wounded area (Nishizaki et al, 1990; Eschwège et al, 1995; Hansen et al, 1995; Choy and McCulloch, 1996). In our experiments the risk of shedding or dislodgement of tumour cells should have been relatively large at the time of implantation or treatment. Manipulation of the tumour was more pronounced during laser thermotherapy than during liver resection, and there was a large incidence of intraperitoneal spread in rats undergoing sham laser therapy (Tables 2 and 3). A relatively small shedding of tumour cells, at the time of treatment, into the peritoneal cavity is therefore an unlikely explanation for the lower incidence of intraperitoneal spread in rats receiving laser thermotherapy than in rats undergoing resection of the tumour-bearing liver lobe. Owing to its ability to seal vessels, it is possible

© Cancer Research Campaign 1998

that laser thermotherapy gives less intravascular tumour cell spread than surgical resection.

Surgery has been shown to cause generalized immunosuppression, including depressed function of immune cells, such as lymphocytes, NK cells and non-parenchymal cells of the liver, which have the capacity to kill tumour cells (Pearson et al, 1986; Pollock et al, 1987, 1992; Colachio et al, 1994; Oka et al, 1994). Surgical resection also induces the production and release of growth factors, events that may stimulate cell division of tumours and facilitate recurrence and spread (Goustin et al, 1986; Fisher et al, 1989; Davies et al, 1994). The role of growth factors may be particularly important after liver resection because of the growth factors produced by the regenerating liver (Loizidou et al, 1991; Mizutani et al, 1992; Jiang et al, 1993). Local accumulation of growth factors and paracrine regulation of tumour growth may play a role for hepatic recurrence (Jiang et al, 1993; de Jong et al, 1995). Promotion of extrahepatic tumour growth after liver resection has been demonstrated in some (Paschkis et al, 1955; Namieno et al, 1991), but not all (Gershbein, 1963; de Jong et al, 1995), studies. Davies et al (1994) reported that antibodies against circulating host-derived epidermal growth factor (EGF) affected the growth of small tumour implants within the peritoneal cavity but not larger, subcutaneous implants. Thus, even if growth factors released systemically after liver resection do not affect all kinds of extrahepatic tumour growth, it is possible that they played a role for the intraperitoneal tumour spread in the present study.

The incidence and extent of extrahepatic spread was not higher in rats treated with resection of the median liver lobe than in rats undergoing sham laser or sham liver resection treatments. Part of the explanation is, perhaps, that a single liver lobe resection is a relatively minor procedure in the rat, although Slooter et al (1995) reported that resection of the left lateral lobe was enough to produce enhancement of tumour growth in the liver. Nevertheless, these findings indicate that the immunosuppressive effects of surgery or the release of growth factors was not the main factor for the increased extrahepatic spread after resection compared with laser thermotherapy.

Tissue breakdown products obtained after local tumour destruction may give rise to a favourable increase of the immune defence (Dickson and Shah, 1982). Cryotherapy of liver cancer has been shown to reduce the risk of metastatic spread as compared with excision in a rat liver tumour model, an outcome that was considered to be due to an immunological response (Jacob et al, 1984). This view was corroborated by the demonstration of resistance to re-challenge after cryodestruction of a subcutaneously inoculated, 3-methylcholanthrene-induced breast adenocarcinoma in the rat (Misao et al, 1981; Miya et al, 1986). In this context, it is interesting that intraperitoneal spread was also reduced after suboptimal laser thermotherapy or in a tumour cell injection model (Table 3). Injection of a suspension of tumour cells gives a more pure antigenic challenge (less contamination by stroma and blood cells), although is less reproducible, at least in our hands.

After hyperthermic treatment there are a number of events that favour an immunological response, including delayed cell death (Fajardo et al, 1980), increased permeability of tumour vessels (Lefor et al, 1985), increased expression of cancer antigens (Wong et al, 1989), increased expression of major histocompatibility complex (MHC) class II antigens (Rees et al, 1991) and increased lymphocyte adhesion to endothelial cells (Lefor et al, 1994). Many tumours express specific antigens that may be presented to T cells by MHC class I molecules (Kovacsovics-Bankowski et al, 1993; Huang et al, 1994; Falo et al, 1995). Heat has been shown to induce increased synthesis and surface expression of heat shock proteins (HSPs) in tumour cells (Multhoff et al, 1995; Wei et al, 1996). Recent studies have suggested that HSPs are involved in eliciting immunity against tumours. HSP70-associated peptides can act as specific immunogenic determinants in tumour cells (Udono and Srivastava, 1993) and HSPs can bind peptides, including tumour-specific peptides, for antigen presentation (Tamura et al, 1997). It has been suggested that HSPs function as carriers of tumour-associated antigenic peptides and that the antigen-HSP complex is taken up by macrophages, which in turn elicits an immune response via MHC class I on the macrophages (re-presentation of the antigen) (Srivastava et al, 1994). Our results after suboptimal thermotherapy and reimplantation of viable tumour (Table 3) are consistent with, but do not prove, involvement of immunological mechanisms. In ongoing experiments, we have found increased expression of HSP70 in tumour cells after interstitial laser thermotherapy, which may be of significance because Tamura et al (1997) showed that autologous cancer-derived HSP-peptide complexes are efficient in cancer therapy.

In conclusion, this study showed that interstitial laser thermotherapy reduces the spread of an experimental liver tumour compared with resection. This finding warrants further study.

### ACKNOWLEDGEMENTS

This work was supported by grants from the Swedish Cancer Society (project no. 3163-B95-03XAA), the Swedish Medical Research Council (project no. B95-17X-07183), the Crafoord Foundation; Lund University; Gunnar, Arvid and Elisabeth Nilsson Foundation for Cancer Treatment, John and Augusta Persson Foundation for Scientific Medical Research, the Royal Physiographic Society in Lund and Bergþóra Magnúsdóttir and Jakob J Bjarnason Memorial Foundation.

### REFERENCES

Amin Z, Donald JJ, Masters A, Kant R, Steger AC, Bown SG and Lees WR (1993) Hepatic metastases: interstitial laser photocoagulation with real-time US monitoring and dynamic CT evaluation of treatment. *Radiology* **187**: 339–347

August DA, Sugarbaker PH, Ottow RT, Gianola FJ and Schneider PD (1985) Hepatic resection of colorectal metastases: influence of clinical factors and adjuvant intraperitoneal 5-fluorouracil via Tenckhoff catheter on survival. *Ann Surg* **201**: 210–218

Bengmark S and Tranberg K-G (1984) Liver physiology and liver resection. In *Surgical Care*, Condon RE and DeCosse JJ. (eds), pp. 93–107. Lea & Febiger: Philadelphia

Bosman S, Phoa SSK, Bosma A and Van Gemert MJC (1991) Effect of percutaneous interstitial thermal laser on normal liver of pigs: sonographic and histopathologic correlations. *Br J Surg* **78**: 572–575

Carlsson G, Gullberg B and Hafström L (1983) Estimation of liver tumor volume using different formulas: an experimental study in rats. *J Cancer Res Clin Oncol* **105**: 20–23

Choy A and McCulloch P (1996) Induction of tumour cell shedding into effluent venous blood breast cancer surgery. *Br J Cancer* **73**: 79–82

Colacchio TA, Yeager MP and Hildebrandt LW (1994) Perioperative immunomodulation in cancer surgery. *Am J Surg* **167**: 174–179

Davies DE, Farmer S, White J, Senior PV, Warnes SL and Alexander P (1994) Contribution of host-derived growth factors to in vivo growth of a transplantable murine mammary carcinoma. *Br J Cancer* **70**: 263–269

De Jong KP, Lont HE, Bijma AM, Brouwers MAM, De Vries EGE, Van Veen ML, Marquet RL, Slooff MJH and Terpstra OT (1995) The effect of partial

© Cancer Research Campaign 1998 

hepatectomy on tumor growth in rats: in vivo and in vitro studies. *Hepatology* **22**: 1263–1272

Dickson JA and Shah SA (1982) Hyperthermia: the immune response and tumor metastasis. *Natl Cancer Inst Monogr* **61**: 183–192

Dingemans KP, Zeeman-Boeschoten IM, Keep RF and Das PK (1993) Transplantation of colon carcinoma into granulation tissue induces an invasive morphotype. *Int J Cancer* **54**: 1010–1016

Eggermont AMM, Steller EP and Sugarbaker PH (1987) Laparotomy enhances intraperitoneal tumor growth and abrogates the antitumor effects of interleukin-2 and lymphokine-activated killer cells. *Surgery* **102**: 71–78

Eschwège P, Dumas F, Blanchet P, Le Maire V, Benoit G, Jardin A, Lacour B and Loric S (1995) Haematogenous dissemination of prostate epithelial cells during radical prostatectomy. *Lancet* **346**: 1528–1530

Fajardo LF, Egbert B, Marmor J and Hahn GM (1980) Effects of hyperthermia in a malignant tumor. *Cancer* **45**: 613–623

Falo LD, Kovacsovics-Bankowski M, Thompson K and Rock KL (1995) Targeting antigen into the phagocytic pathway in vivo induces protective tumour immunity. *Nature Med* **1**: 649–653

Farmer DG, Rosove MH, Shaked A and Busuttil RW (1994) Current treatment modalities for hepatocellular carcinoma. *Ann Surg* **219**: 236–247

Fisher B, Gunduz N, Coyle J, Rudock C and Saffer E (1989) Presence of a growth-stimulating factor in serum following primary tumor removal in mice. *Cancer Res* **49**: 1996–2001

Gershbein LL (1963) Transplanted tumor growth and liver regeneration in the rat. *J Natl Cancer Inst* **31**: 521–528

Goustin AS, Leof EB, Shipley GD and Moses HL (1986) Growth factors and cancer. *Cancer Res* **46**: 1015–1029

Hansen E, Wolff N, Knuechel R, Ruschoff J, Hofstaedter F and Taeger K (1995) Tumor cells in blood shed from the surgical field. *Arch Surg* **130**: 387–393

Huang AYC, Golumbek P, Ahmadzadeh M, Jaffee E, Pardoll D and Levitsky H (1994) Role of bone marrow-derived cells in presenting MHC class I-restricted tumor antigens. *Science* **264**: 961–965

Hughes KS, Simon R, Songhorabodi S, Adson MA, Ilstrup DM, Fortner JG et al (1988) Resection of the liver for colorectal carcinoma metastases: a multi-institutional study of indications for resection. *Surgery* **103**: 278–288

Jacob G, Li AKC and Hobbs KEF (1984) A comparison of cryodestruction with excision or infarction of an implanted tumor in rat liver. *Cryobiology* **21**: 148–156

Jiang WG, Hallett MB and Puntis MCA (1993) Hepatocyte growth factor/scatter factor, liver regeneration and cancer metastasis. *Br J Surg* **80**: 1368–1373

Kovacsovics-Bankowski M, Clark K, Benacerraf B and Rock KL (1993) Efficient major histocompatibility complex class I presentation of exogenous antigen upon phagocytosis by macrophages. *Proc Natl Acad Sci USA* **90**: 4942–4946

Lefor AT, Makohon S and Ackerman NB (1985) The effects of hyperthermia on vascular permeability in experimental liver metastasis. *J Surg Oncol* **28**: 297–300

Lefor AT, Foster CE, Sartor W, Engbrecht B, Fabian DF and Silverman D (1994) Hyperthermia increases intercellular adhesion molecule-1 expression and lymphocyte adhesion to endothelial cells. *Surgery* **116**: 214–221

Loizidou MC, Lawrance RJ, Holt S, Carty NJ, Cooper AJ, Alexander P and Taylor I (1991) Facilitation by partial hepatectomy of tumor growth within the rat liver following intraportal injection of syngeneic tumor cells. *Clin Exp Metastasis* **9**: 335–349

Masters A, Steger AC, Lees WR, Walmsley KM and Bown SG (1992) Interstitial laser hyperthermia: a new approach for treating liver metastases. *Br J Cancer* **66**: 518–522

Misao A, Sakata K, Saji S and Kunieda T (1981) Late appearance of resistance to tumor rechallenge following cryosurgery. *Cryobiology* **18**: 386–389

Miya K, Shigetoyo S, Morita T, Niwa H, Takao H, Kida H and Sakata K (1986) Immunological response of regional lymph nodes after tumor cryosurgery: experimental study in rats. *Cryobiology* **23**: 290–295

Mizutani J, Hiraoka T, Yamashita R and Miyauchi Y (1992) Promotion of hepatic metastases by liver resection in the rat. *Br J Cancer* **65**: 794–797

Moertel CG, Fleming TR, Macdonald JS, Haller DG, Laurie JA, Goodman PJ, Ungerleider JS, Emerson WA, Tormey DC, Glick JH, Veeder MH and Mailliard JA (1990) Levamisole and fluorouracil for adjuvant therapy of resected colon carcinoma. *N Engl J Med* **322**: 352–358

Möller PH, Lindberg L, Henriksson PH, Persson BRR and Tranberg K-G (1996) Temperature control and light penetration in a feedback interstitial laser thermotherapy system. *Int J Hyperthermia* **12**: 49–63

Möller PH, Ivarsson K, Stenram U, Radnell M and Tranberg K-G (1997) Interstitial laser thermotherapy of an adenocarcinoma transplanted into rat liver. *Eur J Surg* **63**: 867–870

Multhoff G, Botzler C, Wiesnet M, Muller E, Meier T, Wilmanns W and Issels RD (1995) A stress-inducible 72-kDa heat-shock protein (HSP72) is expressed on the surface of human tumor cells, but not on normal cells. *Int J Cancer* **61**: 272–279

Murthy SM, Goldschmidt RA, Rao LN, Ammirati M, Buchmann T and Scanlon EF (1989) The influence of surgical trauma on experimental metastasis. *Cancer* **64**: 2035–2044

Namieno T, Tackeichi N, Hata Y, Uchino J and Kobayashi H (1991) Kinetic changes of liver regeneration and hepatocellular carcinoma cells after partial hepatectomy in rats. *Gastroenterol Jpn* **26**: 29–36

Nishizaki T, Matsumata T, Kanematsu T, Yasunaga C and Sugimachi K (1990) Surgical manipulation of VX2 carcinoma in the rabbit liver evokes enhancement of metastasis. *J Surg Res* **49**: 92–97

Oka M, Hazama S, Suzuki M, Wang F, Shimoda K, Iizuka N, Wadamori K, Suzuki T and Attwood S (1994) Depression of cytotoxicity of nonparenchymal cells in the liver after surgery. *Surgery* **116**: 877–882

Orr FW and Warner DJA (1987) Effects of neutrophil-mediated pulmonary endothelial injury on the localization and metastasis of circulating Walker carcinosarcoma cells. *Invasion Metastasis* **7**: 183–196

Panis Y, Ribeiro J, Chrétien Y and Nordlinger B (1992) Dormant liver metastases: an experimêntal study. *Br J Surg* **79**: 221–223

Paschkis KE, Cantarow A, Stasney J and Hobbs J (1955) Tumor growth in partially hepatectomized rats. *Cancer Res* **15**: 579–582

Pearson HJ, Anderson J, Chamberlain J and Bell PRF (1986) The effect of Kupffer cell stimulation or depression on the development of liver metastases in the rat. *Cancer Immunol Immunother* **23**: 214–216

Pollock RE, Lotzová E, Stanford SD and Romsdahl MM (1987) Effect of surgical stress on murine natural killer cell cytotoxicity. *J Immunol* **138**: 171–178

Pollock RE, Lotzová E and Stanford SD (1992) Surgical stress impairs natural killer cell programming of tumor for lysis in patients with sarcomas and other solid tumors. *Cancer* **70**: 2192–2202

Rees ADM, Donati Y, Lombardi G, Lamb J, Polla B and Lechler R (1991) Stress induced modulation of antigen-presenting cell function. *Immunology* **74**, 386–392

Scheele J, Stang R, Altendorf-Hofmann A and Paul M (1995) Resection of colorectal liver metastases. *World J Surg* **19**: 59–71

Slooter GD, Marquet RL, Jeekel J and Ijzermans JNM (1995) Tumour growth stimulation after partial hepatectomy can be reduced by treatment with tumour necrosis factor alpha. *Br J Surg* **82**: 129–132

Srivastava PK, Udono H, Blachere NE and Zihai L (1994) Hypothesis: heat shock proteins transfer peptides during antigen processing and CTL priming. *Immunogenetics* **39**: 93–98

Steele GJ and Sjögren HO (1974) Cross-reacting tumor-associated antigen(s) among chemically induced rat colon carcinomas. *Cancer Res* **34**: 1801–1807

Tamura Y, Peng P, Liu K, Daou M and Srivastava PK (1997) Immunotherapy of tumors with autologous tumor-derived heat shock protein preparations. *Science* **278**: 117–120

Tranberg K-G, Möller PH, Hannesson P and Stenram U (1996) Interstitial laser treatment of malignant tumours: initial experience. *Eur J Surg Oncol* **22**: 47–54

Udono H and Srivastava PK (1993) Heat shock protein 70-associated peptides elicit specific cancer immunity. *J Exp Med* **178**: 1391–1396

Van Den Brenk HAS, Burch WM, Orton C and Sharpington C (1973) Stimulation of clonogenic growth of tumor cells and metastases in the lungs by local X-radiation. *Br J Cancer* **27**: 291–306

Van Hillegersberg R, Van Staveren HJ, Kort WJ, Zondervan PE and Terpstra OT (1994) Interstitial Nd:YAG laser coagulation with a cylindrical diffusing fiber tip in experimental liver metastases. *Lasers Surg Med* **14**: 124–138

Wang LQ, Roos G, Andersson B, Renfjärd E, Persson B and Stenram U (1995) Tumour S-phase activity, nucleotide profile and RNA levels after hepatic artery occlusion and reperfusion in an experimental model of secondary liver carcinoma. *Br J Surg* **82**: 963–967

Wei Y-Q, Zhao X, Kariya Y, Fukata H, Teshigawara K and Uchida A (1996) Induction of autologous tumor killing by heat treatment of fresh human tumor cells: involvement of γδ T cells and heat shock protein 70. *Cancer Res* **56**: 1104–1110

Weiss L, Orr FW and Honn KV (1988) Interactions of cancer cells with the microvasculature during metastasis. *FASEB J* **2**: 12–21

Withers HR and Milas L (1973) Influence of preirradiation of lung development of artificial pulmonary metastastases of fibrosarcoma in mice. *Cancer Res* **33**: 1931–1936

Wong JYC, Mivechi NF and Paxton RJ (1989) The effects of hyperthermia on tumor carcinoembryonic antigen expression. *Int J Radiat Oncol Biol Phys* **17**: 803–808

© Cancer Research Campaign 1998